# Antenatal Ultrasound Diagnosis of Congenital High Airway Obstruction Syndrome: A Case Report and Review of Literature

**DOI:** 10.7759/cureus.4772

**Published:** 2019-05-28

**Authors:** Shaista Afzal, Kulsoom Fatima, Mahnaz Ambareen

**Affiliations:** 1 Radiology, Aga Khan University Hospital, Karachi, PAK

**Keywords:** laryngeal atresia, tracheal atresia, chaos, ultrasound, echogenic lungs

## Abstract

Congenital high airway obstruction syndrome (CHAOS) is a rare life-threatening fetal condition resulting from obstruction of the upper fetal airway which may be partial or complete. Prenatal diagnosis is crucial as it usually results in stillbirth or death after delivery if unrecognized. We report a case of CHAOS that was diagnosed prenatally due to characteristic ultrasound features. We also briefly review literature in light of current management options.

## Introduction

Congenital high airway obstruction syndrome (CHAOS) is a rare fetal life-threatening condition with true incidence being unknown. It occurs as a result of congenital obstruction of the fetal airway secondary to laryngeal and/or tracheal atresia, obstructing laryngeal cysts or obstructing tumors of the oropharynx and the cervical region with laryngeal atresia being the most frequent cause [1-2].

Arizawa et al. first reported the prenatal diagnosis of this condition in 1989, and Hedrick et al. coined the term CHAOS for this condition in the year 1994 [3-4]. Failure to recognize this condition in the prenatal period usually results in stillbirth or death after delivery [5-6]. Fortunately, due to improvements in prenatal imaging, this syndrome can be diagnosed by transvaginal ultrasound as early as 15 weeks of gestation. The typical sonographic features of this syndrome are bilateral enlarged hyperechoic lungs, inverted/ flattened diaphragm and dilated airways up to the point of obstruction. Non-immune hydrops and fetal ascites may also be observed.

Early diagnosis of CHAOS is important as now an evolving surgical treatment called EXIT (ex utero intrapartum treatment) procedure is available with the anticipation to improve neonatal outcome [4]. We report a case of CHAOS which was diagnosed based on distinct ultrasound features along with a brief review of the literature.

## Case presentation

**Figure 1:**
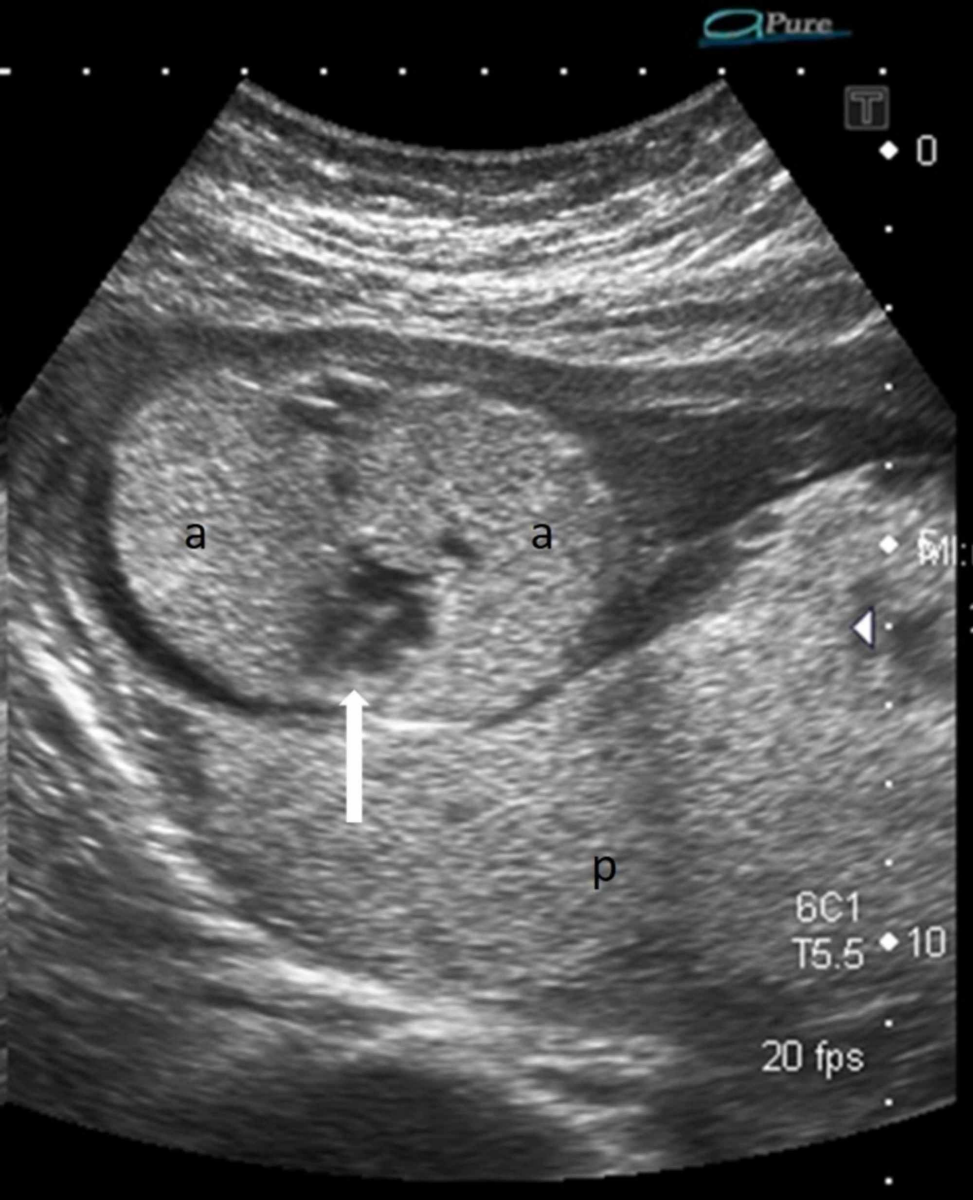
Axial ultrasound of the fetal thorax shows bilateral enlarged echogenic lungs (a) and the centrally placed heart (solid white arrow) Echogenic normal placenta (p) is seen posteriorly; oligohydroamnios is also noted.

A 28-year-old woman, second gravida, presented to the Radiology department for an anomaly scan at 20+ weeks of gestation. The ultrasound showed a single alive fetus with bilateral enlarged echogenic lungs; the heart was compressed by the lungs and was seen centrally within the chest (Figure 1). The four chambers of the heart, however, appeared normal.

**Figure 2:**
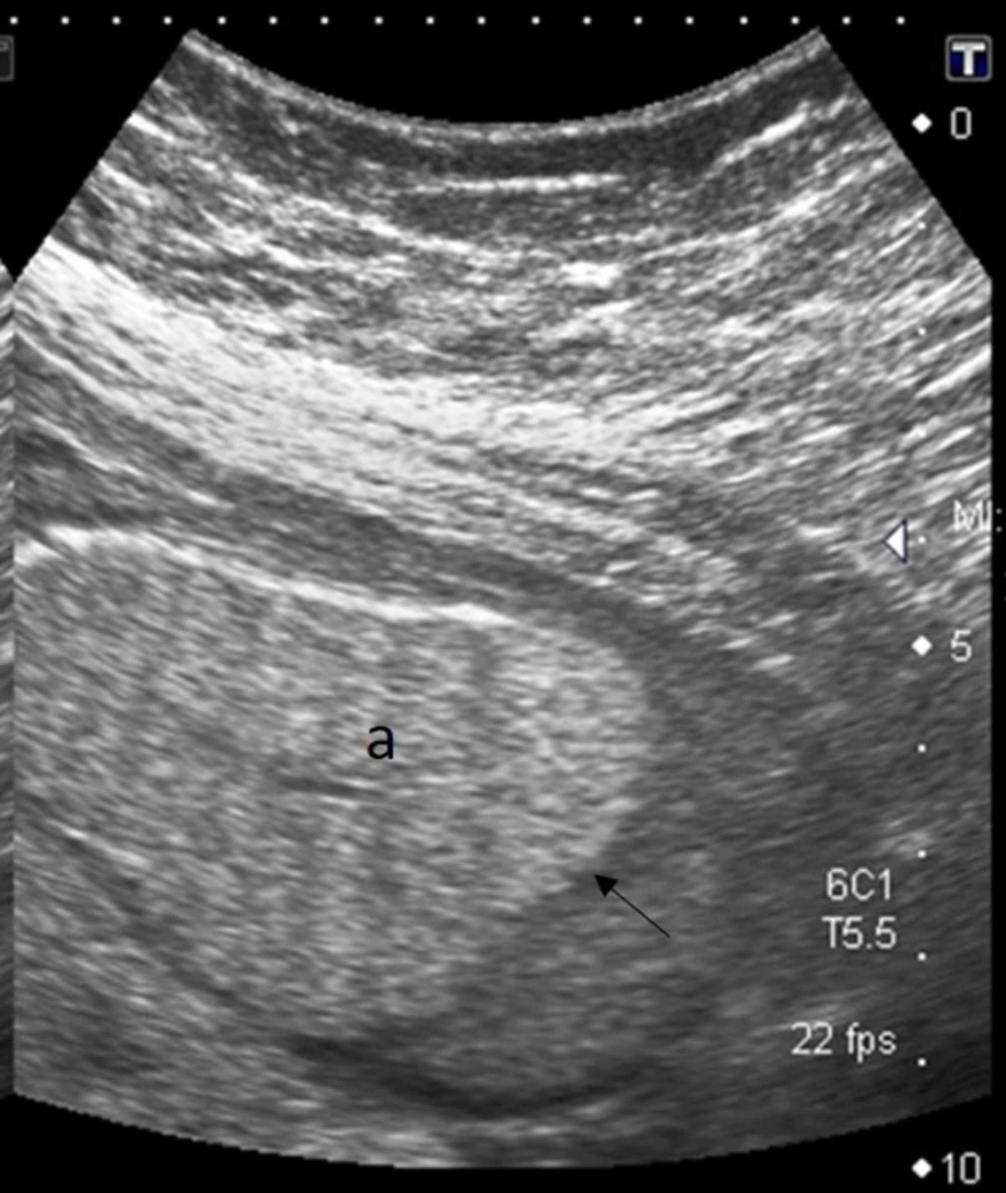
Parasagittal ultrasound image shows inverted diaphragm (black arrow) due to enlarged echogenic lung (a)

Both hemi-diaphragms were inverted (Figure 2).

**Figure 3:**
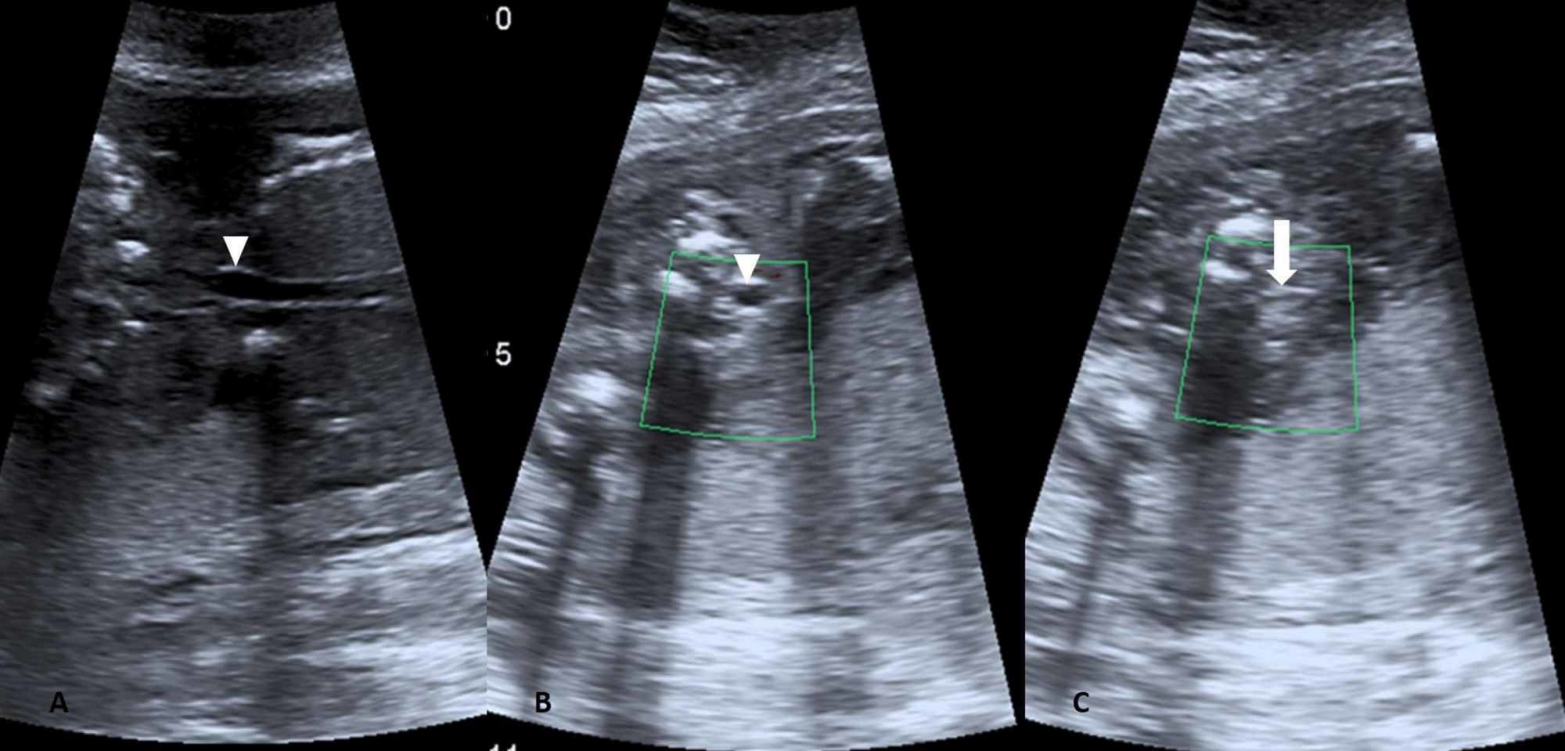
Coronal (A) and axial (B) image show dilated trachea (white inverted triangle), axial image (C) just proximal to it shows the atretic segment (arrow)

**Figure 4:**
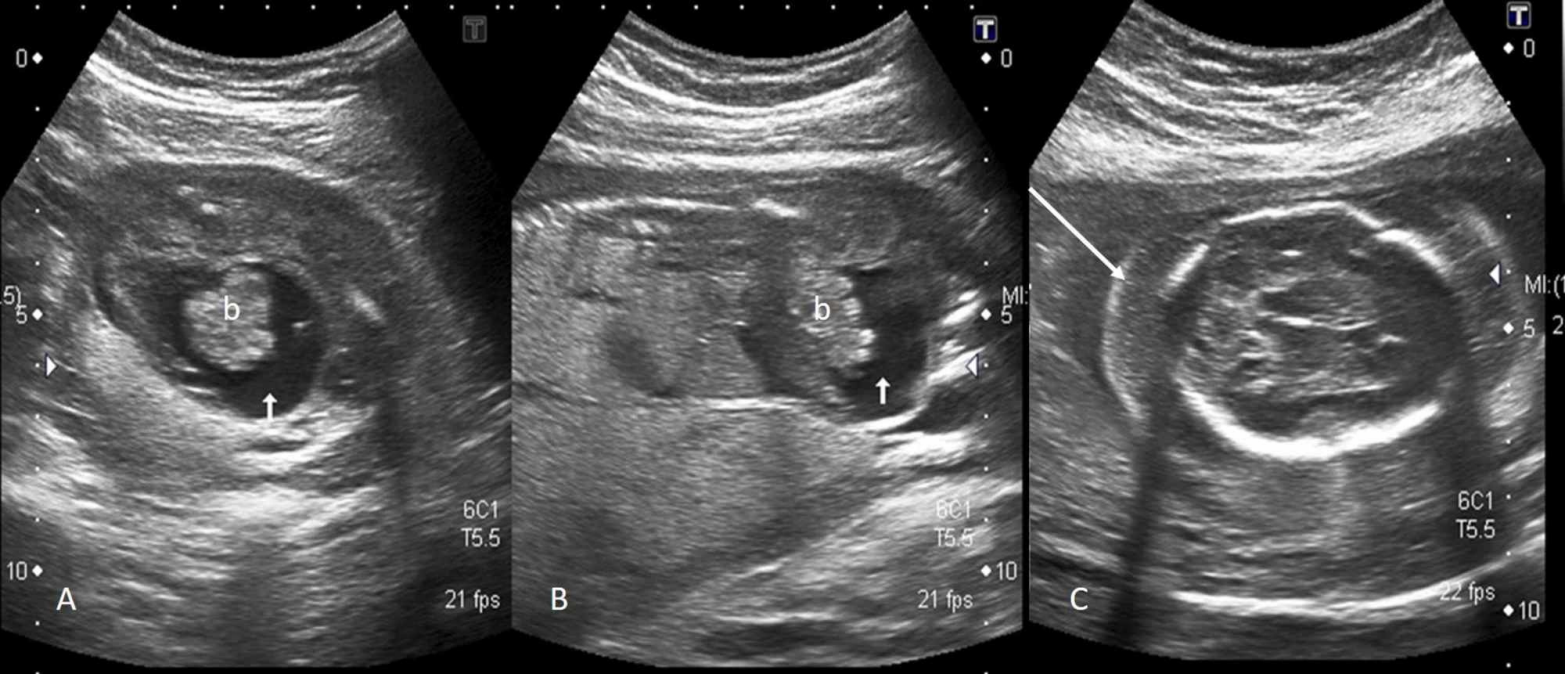
Axial (A) and oblique sagittal (B) image show fetal ascites (short white arrow) and floating bowel loops (b); axial image of fetal head (C) shows significant scalp edema (long white arrow) indicating hydrops fetalis

The trachea was dilated below the level of larynx (Figure 3) and there were fetal ascites and hydrops (Figure 4). The amniotic fluid was reduced with AFI of 7.4 cm which was below the fifth percentile for the gestational age.

The intra-cranial fetal structures, cerebellum, upper lip, spine, both kidneys, stomach bubble, urinary bladder, and the limbs were normal. The placenta was normal appearing. The femur length and bi-parietal diameter corresponded to 19 weeks of gestation while the abdominal circumference was corresponding to 23 weeks due to the presence of ascites. Based on ultrasound findings, the diagnosis of CHAOS due to laryngeal atresia was made.

The patient had a history of one previous miscarriage which occurred at 11 weeks of gestation but no cause was ascertained. There was no history of consanguinity and the family history was also not significant for any congenital disorders.

The parents were counseled by the neonatologist and gynecologist regarding the relatively poor prognosis of this entity and elective termination of pregnancy was undertaken with the parent's consent. Unfortunately, the findings could not be confirmed postnatally as the parents refused postmortem. 

## Discussion

Congenital high airway obstruction syndrome (CHAOS) is a serious fetal abnormality that occurs as a result of deficient recanalization of the upper airway which takes place around the 10th week of gestation and results in laryngeal or tracheal atresia [6-7]. The other rare causes leading to this abnormality are stenosis/atresia of the subglottic region and laryngeal cysts/ webs. The exact incidence of this condition is not known, however, about more than 100 cases have been reported in the literature [1]. The condition is usually sporadic and although no causative gene has been found, about half of the reported cases had several other congenital anomalies including few genetic syndromes like Fraser's syndrome and short rib polydactyly syndrome [1,7]. Therefore, in utero diagnosis of CHAOS necessitates counseling of the couple regarding genetics, further management, and future pregnancies. 

In our case, the trachea was prominent up to the larynx suggesting laryngeal stenosis as the cause of obstruction. In a healthy fetus, the tracheobronchial tree absorbs the fluid secreted by fetal lungs. This fluid, however, cannot be cleared if there is obstruction at the tracheobronchial level and gets accumulated in the fetal lungs, which leads to lung enlargement and on ultrasound results in increased echogenicity of the lungs.

A chain of events ensues i.e. compression of the heart and great vessels by the enlarged lungs leading to its central displacement. The pressure results in reduced cardiac volume and the dysfunctional cardiovascular system leads to fetal hydrops and ascites [8]. Placentomegaly has also been reported in the literature. According to the severity of the process, the diaphragm may be flattened or inverted [7]. In our case, the reported ultrasound features of enlarged echogenic lungs with centrally displaced heart, inverted diaphragm, fetal hydrops, and ascites were noted but there was no placental enlargement. Also, no other congenital abnormality was evident on the anomaly scan.

The first line diagnostic imaging tool is ultrasound due to its ease of availability and affordability. This syndrome can be diagnosed on trans-vaginal ultrasound as early as 15 weeks of gestation. MRI plays an adjunctive role in cases where surgical intervention is planned as it more effectively depicts the dilated airways, the level of obstruction and aids in exclusion of extrinsic pathologies leading to obstruction such as cervical teratoma, lymphatic malformation or vascular rings like double aortic arch [2,9].

In our case, there were no features to suggest the presence of Mirror syndrome in the mother as is reported in the literature and refers to the unusual association of maternal preeclampsia with fetal and placental hydrops. 

The other important differentials for echogenic fetal lungs are congenital cystic adenomatoid malformation (CAM) and pulmonary sequestration (PS). In CAM, the prenatal ultrasound demonstrates the involvement of a lobe of the lung that appears as a hyperechoic mass with the microcysts or an anechoic cyst in case of macrocysts or as a muticystic mass with echogenic stroma in the mixed variety [10]. In PS, an aberrant branch of the aorta directly supplies a portion of lung parenchyma which in most cases has no apparent connection with the airways. The antenatal ultrasound shows a focal uniformly echogenic lung lesion with occasional visualization of arterial supply from the aorta.

Polyhydramnios is usually seen with cases of CHAOS due to compression of esophagus by dilated airways. However, oligohydramnios due to impaired fetal swallowing has also been reported as was evident in our case [6].

With recent advances in neonatal surgery and multidisciplinary approach, cases with partial obstruction and in whom fetal hydrops has not yet set in can be offered EXIT procedure which aims at attaining patent airway before the fetomaternal circulation stops. It entails partial abdominal delivery of the fetal head with the fetal umbilical cord still attached to the placenta and subsequent laryngoscopy and tracheostomy [9]. As ultrasound suggested complete laryngeal obstruction in our case and the fetus had features of hydrops, elective termination of pregnancy was done after counseling the parents.

## Conclusions

Ultrasound is the main diagnostic tool for antenatal diagnosis of congenital high airway obstruction syndrome. The typical ultrasound features help in differentiating this fatal congenital disorder from other non-lethal causes of echogenic fetal lungs. MRI can be offered in selective cases for accurate localization of the level of obstruction where fetal surgical intervention is planned.

Early prenatal diagnosis of CHAOS is essential to either perform elective termination of pregnancy or, in selected cases, at tertiary care centers undergo perinatal surgical management, thereby avoiding post-delivery fetal death or other complications.
